# Contemporary Neurovascular Guidelines Between Historical Evidence and Technological Reality

**DOI:** 10.1007/s00062-026-01682-0

**Published:** 2026-06-03

**Authors:** M. Bendszus

**Affiliations:** Neurologische Klinik, Abteilung für Neuroradiologie, Universität Heidelberg, INF 400, 69120 Heidelberg, Germany

Recent European guidelines on aneurysmal subarachnoid hemorrhage represent an important effort toward standardization of neurovascular care [1]. However, several recommendations concerning contemporary endovascular treatment strategies also highlight broader methodological challenges regarding the interpretation and weighting of evidence across treatment modalities over time.

Modern endovascular techniques such as balloon-assisted coiling (BAC) and intrasaccular flow disruption devices are frequently evaluated against exceptionally high evidentiary thresholds despite being supported by prospective multicenter registries, pooled analyses, and core lab-adjudicated studies demonstrating favorable safety and efficacy profiles [2–5]. In contrast, historical surgical paradigms and concepts originating from the early coiling era continue to exert substantial influence on current these recommendations [1].

This imbalance is particularly evident in the interpretation of BAC-related evidence. Two early single-center reports from the initial adoption era of balloon remodeling—the studies by Sluzewski et al. and Henkes et al.—receive substantial interpretative emphasis despite important methodological limitations including small sample size, heterogeneous procedural workflows, first-generation devices, and considerable selection bias [6, 7]. In contrast, more contemporary positive prospective multicenter evidence such as CLARITY is interpreted more cautiously despite substantially greater methodological robustness and closer relevance to current neurointerventional practice [2].

A similarly illustrative example concerns the interpretation of intrasaccular flow disruption devices. The guideline states that available studies lacked core laboratory adjudication; however, prospective multicenter studies such as CLARYS and WEB-IT did in fact include independent core laboratory assessment of angiographic outcomes as well as independent clinical event adjudication [3, 4]. At the same time, the guideline emphasizes the absence of very long-term follow-up data for WEB treatment despite the fact that the technology has not yet been in sufficiently widespread clinical use to realistically permit robust 10-year outcome analyses. Nevertheless, prospective multicenter studies, pooled analyses, and mid-term follow-up data consistently demonstrate favorable safety and efficacy profiles for intrasaccular flow disruption devices with a follow-up up to 5 years [3–5]. The simultaneous limited consideration of contemporary core lab-adjudicated evidence while emphasizing the absence of very long-term follow-up may reflect differing evidentiary expectations applied to evolving endovascular technologies.

The continued reliance on ISAT-era evidence as a universal benchmark for current endovascular therapy also deserves reconsideration [8]. ISAT fundamentally transformed aneurysm treatment; however, direct extrapolation of outcomes derived from early coil-generation technology to contemporary device-assisted neurointervention introduces substantial temporal evidence bias. Modern aneurysm therapy increasingly incorporates balloon remodeling, intrasaccular flow disruption, advanced imaging guidance, improved microcatheter technology, and optimized neurocritical care pathways that were not represented within the original ISAT framework.

Another illustrative example for weighting evidence across treatment modalities is the guideline recommendation suggesting that patients younger than 40 years should also be considered for microsurgical clipping [1]. This recommendation appears to be influenced substantially by subgroup analyses derived from ISAT-era data, particularly the publication by Mitchell et al. from 2008 [9]. Importantly, the authors themselves presented these findings cautiously and as hypothesis-generating rather than definitive. The willingness to derive contemporary treatment recommendations from retrospective subgroup observations originating more than two decades ago contrasts with the comparatively restrictive interpretation often applied to prospective contemporary endovascular datasets, including multicenter core lab-adjudicated studies.

Importantly, the interpretation of contemporary endovascular techniques within the current European guideline contrasts with the more flexible and individualized treatment framework adopted by the recent American Heart Association/American Stroke Association guideline [10]. The latter explicitly reflects the ongoing evolution of device-assisted neurointervention rather than anchoring recommendations predominantly to historical coiling-era evidence.

Absence of randomized level‑I evidence for newer endovascular technologies should not automatically be interpreted as evidence of inferiority. In rapidly evolving technological disciplines, lack of randomized data often reflects the pace of innovation rather than inadequate safety or efficacy.

Future guideline development may therefore benefit from a more stratified interpretation of evidence incorporating study era, device generation, methodological quality, and technological maturity. Such an approach may better align recommendations with contemporary neurovascular practice while avoiding inadvertent anchoring to technological realities of previous decades.

Guidelines are most valuable when they combine methodological rigor with sufficient adaptability to evolving technological realities. In rapidly innovating fields such as neurointervention, maintaining this balance remains particularly challenging.

## Data Availability

All data supporting the findings of this work are included in the article.
